# MG53 suppresses NF-**κ**B activation to mitigate age-related heart failure

**DOI:** 10.1172/jci.insight.148375

**Published:** 2021-09-08

**Authors:** Xiaoliang Wang, Xiuchun Li, Hannah Ong, Tao Tan, Ki Ho Park, Zehua Bian, Xunchang Zou, Erin Haggard, Paul M. Janssen, Robert E. Merritt, Timothy M. Pawlik, Bryan A. Whitson, Nahush A. Mokadam, Lei Cao, Hua Zhu, Chuanxi Cai, Jianjie Ma

**Affiliations:** 1 Department of Surgery, Division of Cardiac Surgery, Davis Heart and Lung Research Institute, The Ohio State University, Columbus, Ohio, USA.; 2 TRIM-edicine, Inc., Columbus, Ohio, USA.; 3 Department of Cancer Biology and Genetics, College of Medicine, and; 4 Department of Physiology and Cell Biology, Davis Heart and Lung Research Institute, The Ohio State University, Columbus, Ohio, USA.

**Keywords:** Aging, Cardiology, Heart failure, Mouse models, NF-kappaB

## Abstract

Aging is associated with chronic oxidative stress and inflammation that affect tissue repair and regeneration capacity. MG53 is a TRIM family protein that facilitates repair of cell membrane injury in a redox-dependent manner. Here, we demonstrate that the expression of MG53 was reduced in failing human hearts and aged mouse hearts, concomitant with elevated NF-κB activation. We evaluated the safety and efficacy of longitudinal, systemic administration of recombinant human MG53 (rhMG53) protein in aged mice. Echocardiography and pressure-volume loop measurements revealed beneficial effects of rhMG53 treatment in improving heart function of aged mice. Biochemical and histological studies demonstrated that the cardioprotective effects of rhMG53 are linked to suppression of NF-κB–mediated inflammation, reducing apoptotic cell death and oxidative stress in the aged heart. Repetitive administration of rhMG53 in aged mice did not have adverse effects on major vital organ functions. These findings support the therapeutic value of rhMG53 in treating age-related decline in cardiac function.

## Graphical abstract

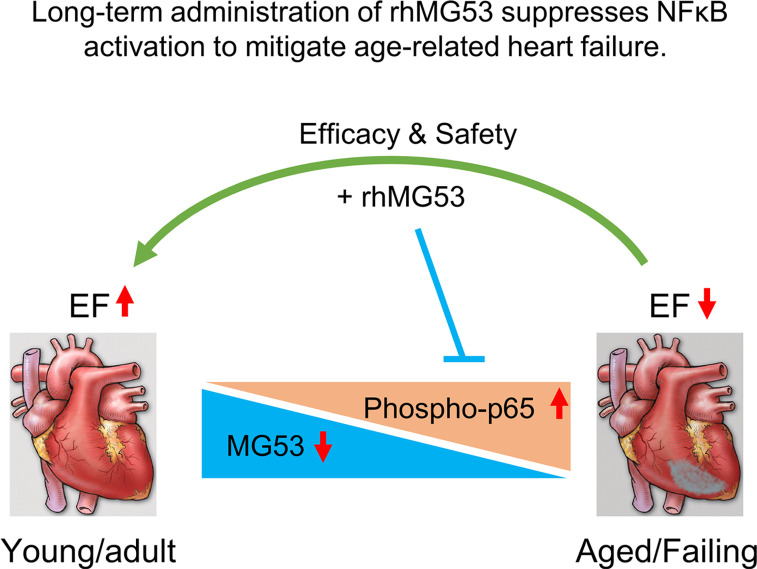

## Introduction

Chronic loss of cardiomyocyte integrity underlies human heart failure (HF) associated with aging that often involves progression of acute myocardial infarction (MI) and the maladaptive response of cardiomyopathy (1). During MI, the membrane repair function of cardiomyocytes is compromised, and protection of membrane integrity is an important strategy to treat MI and HF. In addition, chronic oxidative stress and inflammation associated with aging can render the cardiomyocytes more susceptible to stress-induced MI. Therefore, a therapeutic approach that restores tissue integrity and mitigates inflammation can potentially be an effective means to treat age-related organ dysfunction.

We previously identified MG53 as an essential component of cell membrane repair (2–6). MG53 nucleates the assembly of the membrane repair machinery in a redox-dependent manner. Mice without the MG53 gene develop cardiac pathology due to defective membrane repair and increased susceptibility to cardiac injury (7, 8). Transgenic mice with sustained elevation of MG53 in the bloodstream (~100-fold higher circulating MG53 vs. wild-type mice) lived a healthier and longer lifespan compared with the littermate wild-type mice, and displayed increased tissue healing and regenerative capacity following injury (9). Although we have demonstrated that intravenous administration of recombinant human MG53 (rhMG53) protein could protect against acute heart injury in rodent and porcine models of ischemia-reperfusion–induced MI (5, 10), whether rhMG53 has beneficial effects on chronic HF remains to be determined.

NF-κB is an inflammatory nuclear transcription factor that modulates cardiac function under both physiological and pathophysiological conditions (11, 12). Upregulation of NF-κB is associated with cardiac aging (13), and inhibition of NF-κB signaling in mice displayed beneficial effects for HF, which is related with reduced levels of apoptosis (14) and reactive oxygen species (ROS) production (15–17). Therefore, controlling ROS production has the potential benefit of delaying aging progression. Moreover, recent studies have revealed important crosstalk between inflammation and oxidative stress in the pathogenesis of cardiovascular disease (18, 19). Studies from us and other investigators have suggested a role for MG53 in the control of NF-κB signaling in neuroprotection (20), cardiac hypertrophy (21), and viral infection (22). However, it is not clear how MG53 regulates NF-κB signaling in the aged heart.

In this study, we investigated the expression of MG53 in failing human hearts and aged mouse hearts, and examined whether longitudinal administration of rhMG53 protein in aged mice is safe and exhibits any beneficial effect on the age-related decline in heart function.

## Results

### Expression of MG53 and NF-κB p65 in failing human hearts and aged mouse hearts.

**Figure 1:**
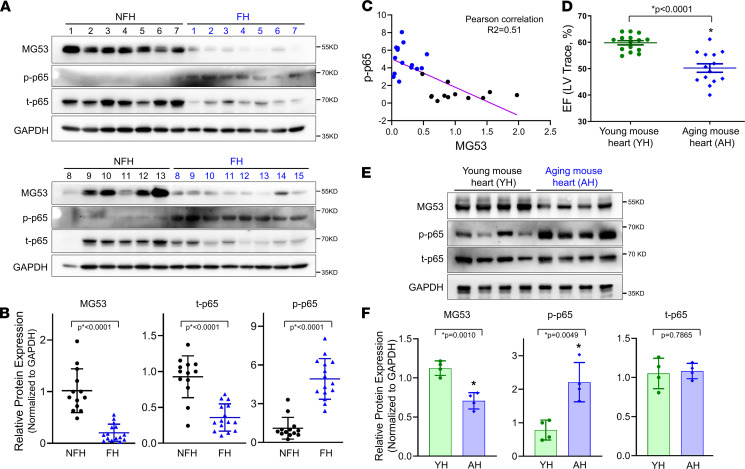
Failing human hearts and aged mouse hearts show reduced MG53 and increased p65 activation. (**A**) Western blot of MG53, total p65 (t-p65), and phosphorylated p65 (p-p65) in nonfailing human hearts (NFHs, *n* = 13) and failing human hearts (FHs, *n* = 15). GAPDH serves as loading control. (**B**) Quantification of MG53, t-p65, and p-p65 in NFH and HF tissues. (**C**) Inverse correlation between MG53 and p-p65 expression in human heart tissues. (**D**) Left ventricular ejection fraction (LV EF) in young (3 months, *n* = 15) and aged (24 months, *n* = 14) mice. (**E**) Western blot for MG53, p-p65, p65 in young mouse hearts (YH, 3 months, *n* = 4) and aged (AH, 24 months, *n* = 4) mouse hearts. (**F**) Quantification of MG53, t-p65, and p-p65 expression in young and aged mouse hearts. Data are expressed as mean ± SEM. Differences were analyzed for significance by unpaired *t* test; *P* values are presented in the individual panels.

A previous study by Lemckert et al. reported low levels of MG53 protein in human hearts (23). We used a custom-made high-affinity antibody that recognizes the human MG53 protein (24) and found that the MG53 protein level was remarkably reduced in the failing human hearts compared with the nonfailing heart, based on Western blotting (Figure 1A). Meanwhile, increased levels of phosphorylated NF-κB p65 (p-p65) were observed in failing human hearts versus nonfailing human hearts (Figure 1, A and B). The demographic information of the human patients is provided in Table 1. The strong correlation between reduced expression of MG53 and elevated activation of NF-κB (Figure 1C) suggests the possibility that the aged human hearts are predisposed to inflammation, which may render then more susceptible to stress-induced cardiac injury.

We obtained aged C57BL/6J mice (24 months of age) from the rodent consortium of the National Institute on Aging. Echocardiography revealed compromised left ventricular (LV) ejection fraction (EF) in the aged versus young mice (3 months age) of the same genetic background (Figure 1D). Similar to the human heart, Western blotting showed that the protein level of MG53 was considerably lower in aged mouse hearts versus young mouse hearts. While the total amount of NF-κB p65 did not appear to change in the aged mouse heart, p-p65 was significantly elevated in the aged mouse hearts compared with the young mouse hearts (Figure 1, E and F).

### Systemic administration of rhMG53 mitigates HF in aged mice.

**Figure 2:**
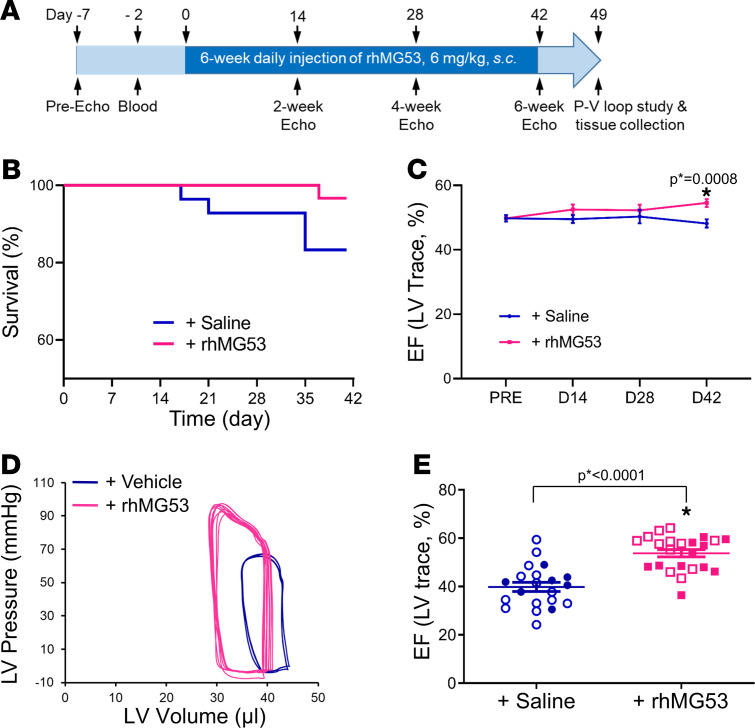
Systemic administration of rhMG53 mitigates HF in aged mice. (**A**) Schematic protocol for the 6-week daily administration of rhMG53 in aged mice. (**B**) Survival curve for mice with systemic administration of rhMG53 (*n* = 30) or saline control (*n* = 30); 15 males and 15 females mice per group. (**C**) Longitudinal echocardiography measurements of aged mice with saline and rhMG53 treatments. (**D**) Representative pressure-volume loop for the assessment of aged mouse heart function after treatment with saline or rhMG53. (**E**) Quantitative analysis of the left ventricular EF for the PV-loop measurements for mice treated with saline and rhMG53. Open symbols, male mice; closed symbols, female mice. Data are expressed as mean ± SEM. Differences were analyzed for significance by unpaired *t* test (**C** and **E**); *P* values are presented in the individual panels.

We designed a protocol to evaluate the safety and therapeutic potential with longitudinal administration of rhMG53 in aged mice (Figure 2A). A total of 60 C57BL/6J mice were examined (24–25 months old, 30 male and 30 female). Half of the mice received saline and half received rhMG53 (6 mg/kg, subcutaneous, daily for 6 weeks). The experiment and data analyses were conducted blindly. Repetitive rhMG53 administration did not produce adverse effects in aged animals, as only 1 out of 30 rhMG53-treated mice died during the 6-week treatment, whereas 5 out of 30 mice died in the saline control group (Figure 2B). Mouse cardiac function was monitored longitudinally using echocardiography, which revealed progressive improvement in LV EF in animals treated with rhMG53 versus those treated with saline (Figure 2C). After 6 weeks of rhMG53 treatment, both male and female mice exhibited significant improvement in LV EF compared with saline treatment. At the end of the 6-week treatment period, terminal pressure-volume loop (PV-loop) assessment was performed to evaluate the changes in contractile function of the mouse heart. As shown in Figure 2D, a clear leftward shift of the PV-loop was observed in the aged mice receiving rhMG53 versus saline control, indicating an improvement in heart function. Quantitative analyses of the PV-loop measurement demonstrated significant improvement in LV EF in aged mice that received the longitudinal rhMG53 treatment (Figure 2E).

### Longitudinal administration of rhMG53 is cardioprotective in aged mice.

**Figure 3:**
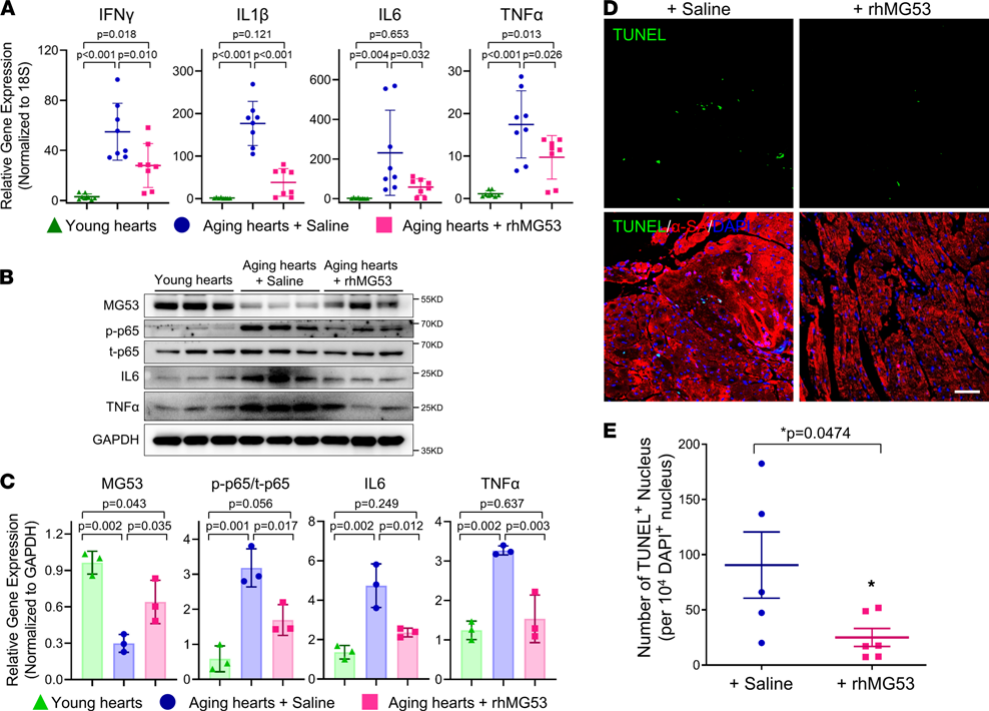
Longitudinal treatment with rhMG53 suppresses activation of the NF-κB signaling pathway and cardiac cell death. (**A**) Quantitative real-time PCR analysis showing the relative mRNA expression of proinflammatory marker genes *Ifng* (IFN-γ), *Il1b* (IL-1β), *Il6* (IL-6), and *Tnfa* (TNF-α), in cardiac muscle collected from young mice (YH, 3 months) and aged mice (AH, 25.5 months) treated with saline or rhMG53 (*n* = 8 per group). (**B** and **C**) Western blot and quantitative analysis of MG53, phosphorylated and total p65, IL-6, TNF-α, and GAPDH (as the loading control) in heart tissues derived from young mice and aged mice treated with saline or rhMG53 (*n* = 3). (**D** and **E**) Representative confocal images and quantitative analysis for the TUNEL staining of heart sections from aged mice treated with rhMG53 (*n* = 6) or saline (*n* = 5). Scale bar: 100 μm. Data are expressed as mean ± SEM. *P* values were calculated using 1-way ANOVA with Tukey’s multiple comparison test (**A** and **C**) or an unpaired *t* test (**E**) and are presented in the individual panels.

Real-time PCR was conducted for cardiac tissue samples collected from the above animal studies to quantify the changes in NF-κB–related signaling components. As shown in Figure 3A, we found increased gene expression of *Ifng* (IFN-γ), *Il1b* (IL-1β), *Il6* (IL-6), and *Tnfa* (TNF-α) in the aged mouse hearts (25 months old) compared with the young mouse hearts (3 months old). Following the 6-week rhMG53 treatment, these myocardial inflammatory genes in the aged mouse heart were significantly diminished when compared with those receiving saline as control (Figure 3A).

We next conducted biochemical assays to determine the signaling machinery that potentially contributes to the cardioprotective effect of rhMG53 in the aged heart (Figure 3, B and C). First, compared with the young mouse hearts, we found that the elevated level of p-p65 in aged mouse hearts was significantly reduced by the administration of rhMG53. The cardioprotective effect of rhMG53 appeared to be linked to suppression of NF-κB–mediated inflammation in the aged mouse heart, where the p-p65/total p65 ratio changed from 4.15 ± 0.35 (+saline) to 2.27 ± 0.34 (+rhMG53) (*P* < 0.01, *n* = 3). Second, we observed that the reduced expression of endogenous MG53 protein in the aged mouse heart was partially restored after the 6-week period of repetitive rhMG53 treatment. Since the Western blot was performed 1 week after the last rhMG53 administration (when mice were sacrificed and hearts isolated), it is unlikely that the increased MG53 protein originated from the exogenous rhMG53. Third, we found that the protein expression of IL-6 and TNF-α, downstream NF-κB–regulated proinflammatory markers, was higher in hearts derived from aged mice receiving saline, which is consistent with other investigators (18, 19), and rhMG53 treatment ameliorated the elevated expression of IL-6 and TNF-α in the aged mouse heart (Figure 3, B and C).

TUNEL staining was performed with sections of the mouse heart to evaluate the potential benefits of rhMG53 in preserving cardiomyocyte integrity in the aged mice. As shown in Figure 3, D and E, mouse hearts with rhMG53 treatment exhibited significantly fewer TUNEL-positive cells versus the saline control group. Overall, these data suggest that the multicellular function of rhMG53 in cardioprotection is associated with antiinflammation and reduced apoptotic cell death in the aged heart.

### Effects of rhMG53 treatment on oxidative stress response in aged hearts.

**Figure 4:**
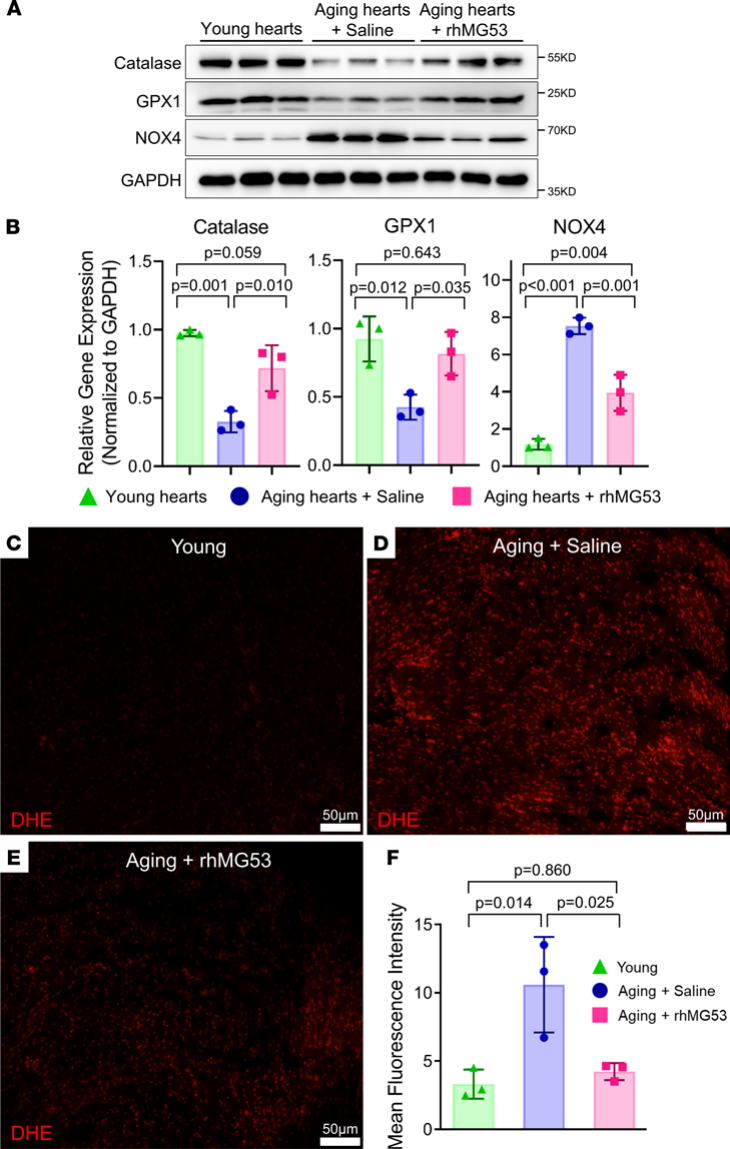
Daily administration of rhMG53 regulated the expression of oxidative stress–associated proteins and suppressed age-related ROS generation. (**A** and **B**) Western blot and quantitative analysis of catalase, GPX1, NOX4, and GAPDH (as the loading control) in heart tissues derived young mice (3 months) and aged mice treated with saline or rhMG53 (25.5 months). The samples and loading condition were identical to those in Figure 3B. (**C**–**E**) Cross sections of mouse left ventricles stained with dihydroethidium (DHE) for quantification of ROS in young mice (**C**) and aged mice treated with saline (**D**) or rhMG53 (**E**) (*n* = 3). Scale bars: 50 μm. (**F**) The quantification of the data in **C**–**E**. Data are expressed as mean ± SEM. *P* values were calculated using 1-way ANOVA with Tukey’s multiple comparison test (**B** and **F**) and are presented in the individual panels.

We also investigated the activity of antioxidant and oxidant proteins in aged mouse hearts treated with saline or rhMG53. As shown in Figure 4, A and B, the levels of catalase and GPX1, known antioxidant proteins, were significantly lower in saline-treated aged mouse hearts compared with the young mouse hearts, whereas the protein level of NADPH oxidase 4 (NOX4), which is thought be responsible for oxidative stress, was considerably higher in saline-treated aged mouse hearts compared with the young mouse hearts (Figure 4, A and B). rhMG53 treatment restored the expression of catalase and GPX1, and decreased the expression of NOX4 in the aged mouse hearts. These findings indicated that rhMG53 treatment could reduce oxidative stress in the aged mouse heart.

Dihydroethidium (DHE) fluorescence staining was conducted to determine the level of ROS in the mouse hearts. The DHE fluorescence intensity was lower in young mouse hearts (Figure 4C) compared with the aged mouse hearts receiving saline treatment (Figure 4D). The aged mice receiving the 6-week longitudinal treatment with rhMG53 showed significant reduction in DHE fluorescence intensity (Figure 4E). Data from multiple animals are summarized in Figure 4F. Collectively, these data suggest that longitudinal treatment with rhMG53 suppressed the age-induced oxidative stress response in the heart.

### Repetitive administration of rhMG53 is safe in aged mice.

Over the 6-week rhMG53 treatment period, each mouse received a total of approximately 6 mg rhMG53 protein. If there were any immune response or toxicity with the repetitive rhMG53 administration in the aged mice, this should be reflected in the vital organs, including the heart, liver, and spleen. Our previously published data (25) showed that young, healthy rats treated with repetitive administration of rhMG53 (up to 40 mg/kg) did not show any sign of cardiotoxicity or adverse effects. We did not expect that repetitive rhMG53 administration would have any side effects in healthy young mice; thus, we did not include a group of young mice treated with rhMG53 in the present study.

**Figure 5:**
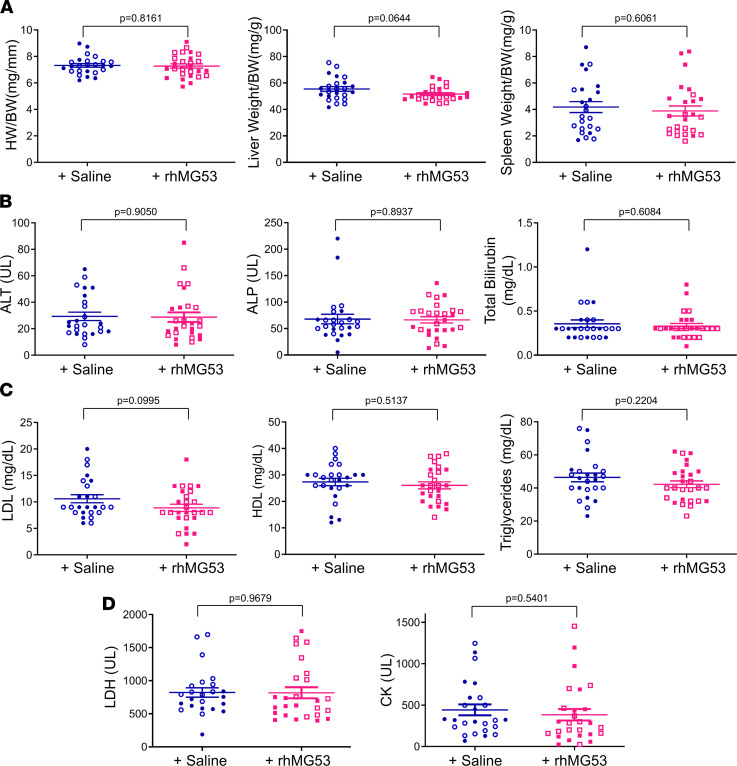
Repetitive administration of rhMG53 is safe in aged mice. (**A**) The ratios of heart weight/tibia length (HW/TL), liver weight/body weight (BW), and spleen weight/BW were not significantly changed after 6-week administration of rhMG53 in aged mice versus the saline group. (**B**) Serum levels of ALT, ALP, and total bilirubin in mice treated with saline or rhMG53. (**C**) Serum levels of LDL, HDL, and triglycerides in mice treated with saline or rhMG53. (**D**) Serum levels of LDH and CK in mice treated with saline or rhMG53. Open symbols, male mice; closed symbols, female mice. Data are expressed as mean ± SEM. Differences were analyzed for significance by an unpaired *t* test (**A**–**D**); *P* values are presented in the individual panels.

After sacrificing the animals at the end point, the heart weight (HW) and tibia length (TL) were measured. The HW/TL ratios of individual mice were plotted and are shown in Figure 5A. Clearly, there was no change in HW/TL with or without rhMG53 treatment. Echocardiographic analyses showed no significant differences in diastolic and systolic LV anterior wall thickness or posterior wall thickness in aged mice treated with rhMG53 or saline (Supplemental Figure 1; supplemental material available online with this article; https://doi.org/10.1172/jci.insight.148375DS1), suggesting that repetitive rhMG53 administration did not induce hypertrophy or hypotrophy in the aged mouse heart. Meanwhile, the ratios of the liver weight over the body weight and the spleen weight over the body weight did not appear to be different between the mice receiving rhMG53 or saline (Figure 5A). We also quantified the serum levels of alanine aminotransferase (ALT), alkaline phosphatase (ALP), and total bilirubin (TBILI) as biomarkers for liver injury and dysfunction (Figure 5B), and found no significant differences between the 2 groups of aged mice. Furthermore, the serum contents of high-density lipoprotein (HDL), low-density lipoprotein (LDL), and triglycerides were also quantified (Figure 5C). Additionally, we quantified the serum levels of lactate dehydrogenase (LDH) and creatine kinase (CK), and found no significant difference between the saline control and rhMG53 treatment groups (Figure 5D). Overall, there were no measurable changes in ALT, ALP, TBILI, LDL, HDL, triglycerides, LDH, and CK in aged mice following the 6-week treatment with rhMG53, suggesting no adverse effects of rhMG53 on hepatic and cardiovascular functions. These data support the conclusion that systemic and repetitive rhMG53 administration is safe in aged mice.

## Discussion

Elderly people have an increased risk for MI and HF due to compromised tissue repair and regeneration. In this study, we show that the expression of MG53 is decreased in both the failing human heart and the aged mouse heart, which is accompanied by elevation of NF-κB activation. Longitudinal administration of rhMG53 mitigated the heart dysfunction in the aged mice. We demonstrate that repetitive rhMG53 administration in aged mice is safe and effective in reducing apoptotic cardiomyocyte death and suppressing the chronic inflammation mediated by NF-κB. These findings may provide a basis for the potential use of rhMG53 to treat aging-related HF.

Since our initial discovery of MG53 in 2009, notable progress has been made in advancing the mechanistic action of this gene in the biology of tissue repair as well as the regulation of metabolic syndromes. We know that genetic ablation of MG53 leads to defective cell membrane repair, which can cause progressive skeletal myopathy (2) and reduced survival capacity of cardiomyocytes (7). We recently reported that transgenic mice (tPA-MG53) with sustained elevation of MG53 in the bloodstream lived a healthy lifespan and displayed enhanced tissue regenerative capacity without impairing the body’s metabolic function of glucose handling and insulin signaling (9). In the present study, we demonstrated for the first time to our knowledge that rhMG53 has anti-aging function, e.g., systemic administration of the rhMG53 protein improves survival of the aged mice. We present in vivo data to show that rhMG53 is effective and safe in preventing age-related HF. Our results demonstrated that the aged mice at the end stage of their life (25–27 months age) could tolerate repetitive systemic administration of rhMG53 (6 mg/kg) on a daily basis for 6 weeks. Using a combination of human data and biochemical analyses, we present evidence that the therapeutic benefit of rhMG53 is linked to mitigation of aging-related inflammation and oxidative stress.

Xiao and colleagues proposed that MG53-mediated downregulation of insulin receptor substrate 1 (IRS-1) could serve as a causative factor for the development of type II diabetes (26), but earlier studies from other investigators demonstrated that genetic ablation of IRS-1 is not sufficient to induce type II diabetes in mice (27, 28). Wu et al. recently reported an immune approach using an anti-MG53 antibody that could reduce serum glucose to treat diabetes (29) (see also Zhu et al., ref. 30). We have obtained a substantial amount of data establishing the safety profile of systemic rhMG53 delivery in rodents (31–34), pigs (5), and dogs (33). In particular, the pharmacokinetic properties of rhMG53 in the serum remained unchanged between the beginning and the end of repeated intravenous dosing in dogs (33), suggesting that systemic administration of the human MG53 protein does not produce anti-drug antibody responses in dogs. Data presented in this study further showed that long-term repetitive dosing of rhMG53 is safe and did not alter the liver and cardiovascular function of aged mice.

Increased oxidative stress and apoptosis are associated with the pathogenesis of HF in aging (16, 17). Free radicals generated by ROS are maintained at physiological levels by several endogenous antioxidants, including catalase. A previous study demonstrated that cardiac aging phenotypes were significantly ameliorated by overexpression of catalase in a murine model (16). Overproduction of ROS in aging is known to trigger NF-κB–related inflammatory signaling in cardiomyocytes (12, 13, 15). Our results demonstrated that aging significantly increased the NF-κB–mediated inflammatory response, and decreased the expression of antioxidant proteins in heart tissue, which were partially recovered by the long-term administration of rhMG53, leading to reduced cardiac inflammation, ROS production, and cardiac apoptosis.

We were surprised to see a decrease in total p65 protein in failing human hearts versus nonfailing age-matched human hearts (as shown in Figure 1A). A previous study by Morishita et al. using an NF-κB–binding decoy oligonucleotide to downregulate p65 expression in the rat heart demonstrated that reduced p65 expression had benefits in reducing MI following coronary artery ligation (35). It is possible that decreased total p65 expression might be associated with the adaptation of the progression of HF. Further studies are required to test this possibility.

Some previous studies showed that healthy aged mice do not exhibit significant systolic dysfunction, but not impaired diastolic function. However, most of these studies used mice of a relatively young age, including mice at the age of 6–18 months (36), 18–19 months (37, 38), or 20–24 months (39). However, the mice used in our present study were 24–25 months of age at the start of the rhMG53 (or saline) treatment, and these mice do consistently show reduced LV EF. Our findings are consistent with other published studies showing decreased systolic dysfunction in aged mice (16, 40–42), and rhMG53 treatment improved systolic function of the aged mouse heart. Aging is also associated with an increased proportion of senescent cells, which contribute to inflammation through an increase in the senescence-associated secretory phenotype (43). Whether or not rhMG53 could rejuvenate the senescent phenotype of cardiac cells in aged hearts remains to be examined in the future.

Two limitations were recognized in this study: First, in addition to the NF-κB signaling pathway, there might be other pathways involved in the cardiac action of MG53 in age-related HF. Therefore, future studies with RNA-sequencing analysis could be conducted with aged mouse hearts after systemic treatment with rhMG53 to determine the signaling components involved in cardioprotection. Second, while we have data to correlate the reduced MG53 protein expression and cardiac dysfunction in the aged mouse heart, we do not have a sufficient number of young, healthy human heart samples to establish whether natural aging can also affect the expression of MG53 in healthy human hearts. We have only compared MG53 expression in aged human heart samples from ischemic HF to those without HF. Future studies with sufficient numbers of young healthy human heart samples should examine whether natural aging affects the expression of MG53 in human heart.

Identification of MG53 as a negative regulator of NF-κB, as reflected by the close correlation between reduced MG53 expression and elevated NF-κB activation we observed in both human and mouse hearts, should have broader implications for treatment of other stress-induced heart injuries. For example, the recent outbreak of COVID-19 has caused a devastating global health emergency. While the focus of attention has generally been on pulmonary manifestations, there is emerging evidence that cardiac involvement may be common (44). Patients infected with SARS-CoV-2 have a robust inflammatory response that potentiates the fulminant myocarditis in some patients. Most importantly, cardiac injury occurs more frequently in elderly patients who suffer from SARS-CoV-2 infection and is associated with higher in-hospital mortality (45). While the physiological basis of the susceptibility of the aged heart to virus-induced dysfunction remains largely unknown, a therapeutic approach such as MG53 that mitigates inflammation and restores cardiomyocyte integrity could potentially be an effective means to treat SARS-CoV-2–induced myocarditis in the elderly population. Future studies are required to test whether rhMG53 protein has any therapeutic benefits to treat virus-induced heart injury.

## Methods

### Experimental animals.

The young (3 months old) C57BL/6J mice were purchased from The Jackson Laboratory, and the aged (24–25 months old) mice on the C57BL/6J background were obtained from the National Institute on Aging. rhMG53 (6 mg/kg) or saline was subcutaneously injected once a day for a total of 6 weeks. Immediately after the PV-loop measurement, the serum and vital organs, including the heart, liver, lung, and kidney, were collected for further investigation, as described below. All experiments were conducted in a blinded fashion, e.g., the person who performed data analysis was only given barcode-labeled animals, heart tissues, and serum samples. Archiving and statistical data analyses were conducted by a third person who had no knowledge of the mouse treatment history.

### RNA isolation and quantitative real-time PCR.

Real-time qPCR was performed to determine the proinflammatory cytokine gene expression, including *Ifng*, *Il1b*, *Il6*, and *Tnfa*, for LV tissue collected from young and aged mice treated with saline or rhMG53. The total RNA of each sample was isolated by using the RNeasy Fibrous Tissue Kit according to the manufacturer’s instructions (Qiagen, 74704). The reverse transcription of 1 μg of RNA to cDNA was performed by using the Bio-Rad iScript cDNA Synthesis kit (Bio-Rad, 170-8891). Primer sequences used were *Ifng*-F, CTCTTCCTCATGGCTGTTTCT; *Ifng*-R, TTCTTCCACATCTATGCCACTT; *Il1b*-F, GGTACATCAGCACCTCACAA; *Il1b*-R, TTAGAAACAGTCCAGCCCATAC; *Il6*-F, CTTCCATCCAGTTGCCTTCT; *Il6*-R, CTCCGACTTGTGAAGTGGTATAG; *Tnfa*-F, TTGTCTACTCCCAGGTTCTCT; and *Tnfa*-R, GAGGTTGACTTTCTCCTGGTATG. Samples for real-time qPCR were prepared according to the manufacturer’s instructions in the iTaq Universal SYBR Green Supermix (Bio-Rad, 172-5124), and the reactions were run in a Bio-Rad CFX384 Real-Time system.

### Immunoblotting.

Snap-frozen heart tissues were processed in radioimmunoprecipitation assay (RIPA) lysis buffer (10 mM Tris-HCl, pH 7.2, 150 mM NaCl, 1% NP-40, 0.5% SDS, and 0.5% deoxycolate), supplemented with a cocktail of protease inhibitors (Sigma-Aldrich) and phosphatase inhibitors (Thermo Fisher Scientific for Western blot analyses. Crude extracts from hearts of experimental animals were washed twice with ice-cold PBS and lysed in RIPA buffer plus the same inhibitors. Heart lysates were resolved by 10% SDS-PAGE and transferred onto polyvinylidene fluoride (PVDF) membranes. The blots were washed with Tris-buffered saline with Tween 20 (TBST), blocked with 5% milk in TBST for 1 hour, and incubated with a custom-made monoclonal anti-MG53 antibody (clone 914; refs. 24, 33). Immunoblots were visualized with an ECL Plus kit (Pierce). In addition, antibodies against the following proteins were used in this study: GAPDH (Sigma-Aldrich, CB1001), t-p65 (Cell Signaling Technology, 8242), p-p65 (Cell Signaling Technology, 3033), IL-6 (Cell Signaling Technology, 12153), TNF-α (Cell Signaling Technology, 3707), catalase (Santa Cruz Biotechnology, SC-271803), GPX1 (Thermo Fisher Scientific, PA5-30593), NOX4 (Abcam, ab133303). See complete unedited blots in the supplemental material.

We routinely used multiple samples derived from different animals in a given Western blot. To illustrate the spread of the band intensity with the wild-type samples, we normalized the band intensity to one control sample (which was often the one with mid-range intensity) and plotted the relative intensity of other samples in the scatter plot.

### Echocardiography.

Serial M-mode echocardiographic images of mice subjected to 1%–2% isoflurane anesthesia were obtained via a Vevo 2100 imaging platform (VisualSonics) at baseline (4 days prior to rhMG53 administration), 2 weeks, 4 weeks, and 6 weeks after rhMG53 injection. Using a rectal temperature probe, body temperature was carefully maintained between 36.7°C and 37.3°C throughout the study. Hearts were viewed in the short axis between the 2 papillary muscles. Each measurement was obtained in M-mode by averaging results from 3 consecutive heart beats. LV internal dimensions (LVID) at diastole and systole (LVIDd and LVIDs) were measured. LV EF was calculated using the following formula: EF (%) = 100 × ([LVIDd3 – LVIDs3]/LVIDd3). Digital images were analyzed off-line by blinded observers using the Vevo 2100 workstation software.

### Hemodynamic studies.

At week 7 after the first rhMG53 administration, in vivo cardiac hemodynamic function was evaluated utilizing the Mikro-Tip Pressure Volume System Ultra Foundation Systems (AD Instruments) with a 1.0-French PVR-1045 microtip ultraminiature pressure-volume (PV) catheter (Millar). Mice were anesthetized with 1%–2% isoflurane, intubated, and ventilated with a positive-pressure ventilator (Harvard Apparatus Minivent, Hugo Sachs Electronik; ventilation rate 105/min, tidal volume 10.3 μL/g). Rectal temperature was kept between 36.7°C and 37.3°C. The right common carotid artery was isolated and cannulated (PV catheter) for determination of LV performance. The raw pressure and volume data collected in text files by the MPCU-200 unit and Chart/Powerlab software were imported into the PVAN software, which applied a variety of algorithms to the PV data to calculate up to 30 cardiovascular parameters (18, 19). As in the case of the echo study, all hemodynamic data analyses were performed off-line by investigators blinded to the treatment.

### TUNEL staining.

At the end of the protocol, the heart was arrested in diastole by an intravenous injection of 0.15 mL of CdCl_2_ (100 mM), excised, and perfused retrogradely at 60–80 mmHg (LV end-diastolic pressure = 8 mmHg) with heparinized PBS followed by 10% neutral buffered formalin solution for 15 minutes. The heart was then sectioned into 3 slices from apex to base, fixed in formalin for 24 hours, and subjected to tissue processing, paraffin embedding, and heart sectioning. Paraffin-embedded, 4-μm-thick heart sections were deparaffinized in xylene and rehydrated gradually through 100%, 95%, and 70% ethanol followed by antigen retrieval. After preincubation with serum blocking solution, the primary antibodies were applied to identify the expression of different markers, including anti–α actinin (sarcomeric) antibody (Sigma-Aldrich, A7732) for cardiomyocytes. TUNEL staining was performed to detect apoptotic nuclei by using the terminal deoxynucleotidyltransferase–mediated in situ fluorescein-conjugated dUTP nick-end labeling technique according to the manufacturer’s protocol (GeneCopoeia, VB-4005G). Nuclei were counterstained with DAPI. The fluorescence staining was viewed with a Zeiss 780 confocal laser-scanning microscope. The number of apoptotic cells with TUNEL-positive nuclei is expressed as a percentage of total cells.

### DHE fluorescence staining for the in situ detection of ROS production.

Frozen heart sections were used to examine the levels of ROS production via DHE fluorescence staining. Briefly, the slices with heart cross sections were first incubated with PBS for 10 minutes at 37°C, and then with fresh PBS containing DHE at a final concentration of 5 μM in a light-protected humidified chamber for 30 minutes at 37°C. Images were obtained with a fluorescence microscope (×200 magnification), and the mean fluorescence intensity was quantified by ImageJ (NIH).

### Blood plasma sample collection and analysis.

At the time of sacrificing the mice, whole blood was collected in heparin-treated tubes and centrifuged at 1,000*g* for 15 minutes at 20°C. The upper layer containing blood plasma was then transferred into new tubes for further analysis. The plasma levels of ALT, ALP, TBILI, HDL, LDL, triglycerides, LDH, and CK were evaluated using a Vet Axcel Chemistry Analyzer and Liquid Ready reagents (Alfa Wassermann Diagnostic Technologies) according to the manufacturer’s protocols.

### Statistics.

All of the experimental data were analyzed using GraphPad Prism 8.3 software. The standard error of mean (SEM) is indicated by error bars for each group of data. Data are expressed as the mean ± SEM. Comparisons were made by Student’s *t* test when comparing 2 experimental groups. One-way ANOVA followed by Tukey’s multiple comparison test was used to compare data from 3 different groups. A *P* value of less than 0.05 was considered significant.

### Study approval.

Animal handling and surgical procedures were performed according to protocols approved by the Institutional Animal Care and Use Committee (IACUC) of The Ohio State University and were compliant with guidelines of the American Association for the Accreditation of Laboratory Animal Care. Nonfailing and failing human patient LV tissues were obtained from donor hearts in collaboration with the Lifeline of Ohio Organ Procurement Program (LOOP). Use of these human tissues were approved by the Institutional Review Board (IRB) of The Ohio State University (study number 2012H0197). All participants in these studies provided written informed consent. Snap-frozen heart tissues were processed in radioimmunoprecipitation assay (RIPA) lysis buffer for Western blot analyses.

## Author contributions

XW performed the majority of experiments and analyzed the data. XL, HO, TT, KHP, and ZB participated in some of the experiments and data analysis. XZ and LC provided help with the frozen tissue sectioning. HZ, EH, PMJ, REM, TMP, BAW, and NAM provided valuable suggestions to the study. All authors contributed to editing of the manuscript. CC and JM conceived the study and wrote and revised the manuscript.

## Supplementary Material

Supplemental data

## Figures and Tables

**Table 1:**
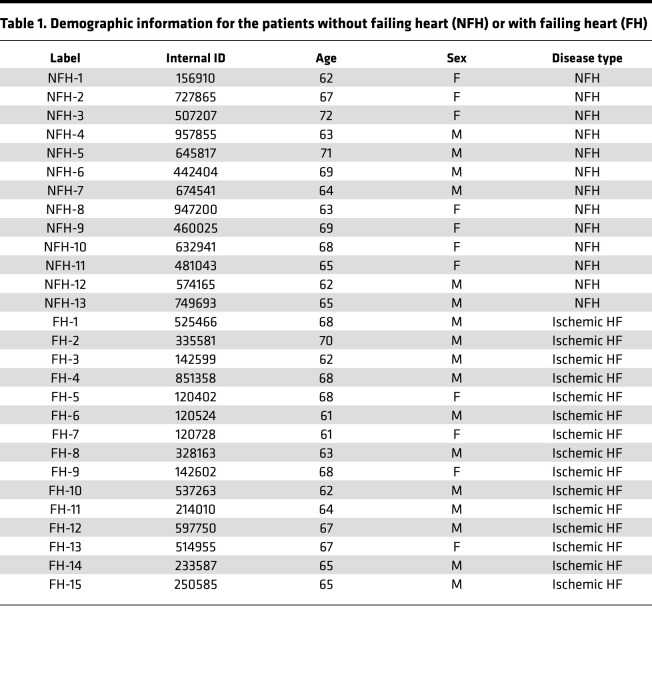
Demographic information for the patients without failing heart (NFH) or with failing heart (FH)

## Version 1 07/22/2021

In-Press Preview

## Version 2 09/08/2021

Electronic publication
