# Supplementary material for: MG53 suppresses NF-κB activation to mitigate age-related heart failure
Source: JCI Insight. 2021 Sep 8;6(17):e148375. doi: 10.1172/jci.insight.148375 (PMC8492351; doi:10.1172/jci.insight.148375)
Supplement: Supplemental data [file jciinsight-6-148375-s098.pdf]

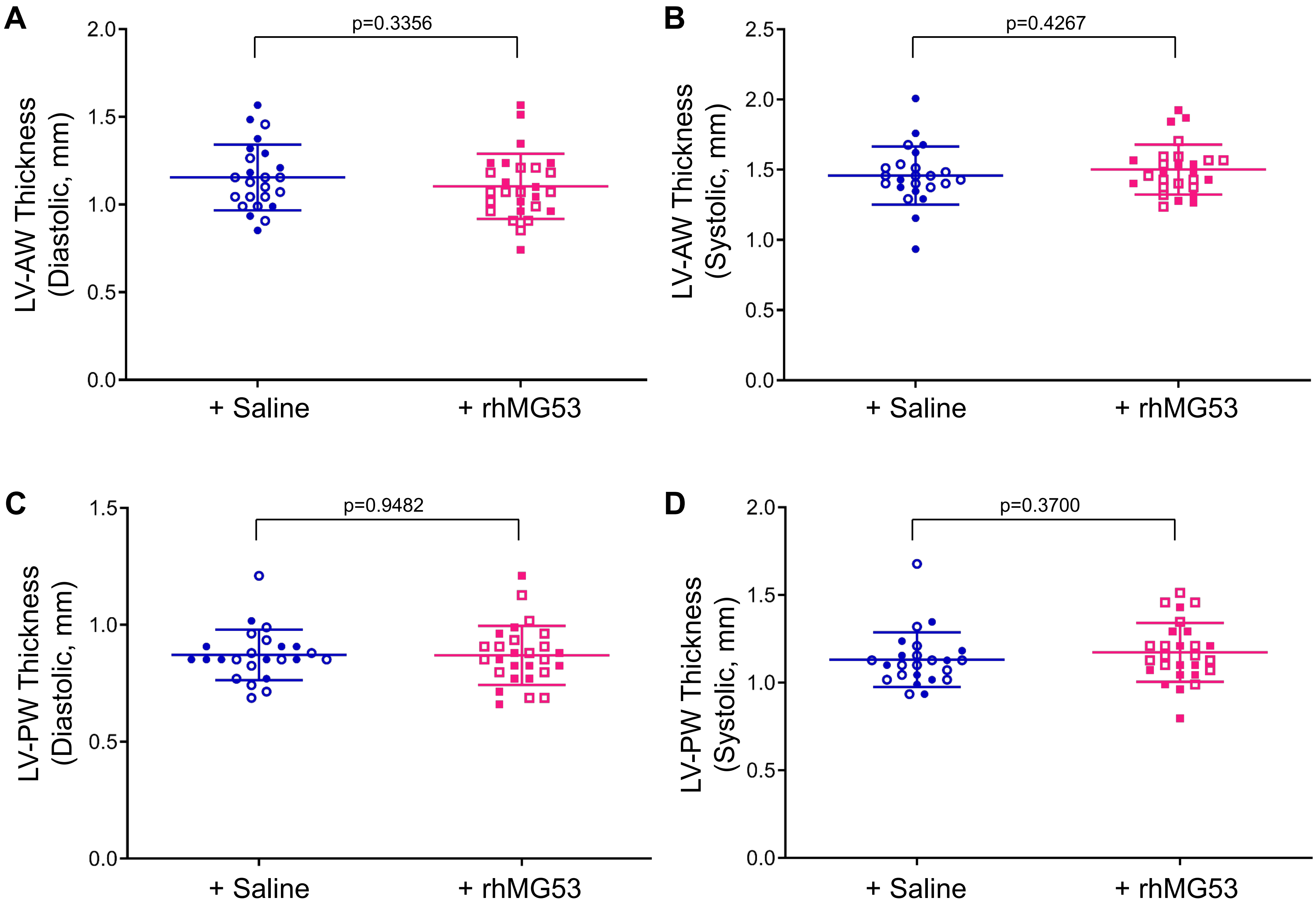

Supplemental Figure S1. Echocardiographic data analysis showed that there are no adverse effects of rhMG53 treatment on the diastolic and systolic LV anterior wall (LV-AW) thickness (**A**&**B**) or the diastolic and systolic LV posterior wall (LV-PW) thickness (**C**&**D**) in aging mice treated with rhMG53 or saline. Open symbols - male mice; closed symbols - female mice. Data are expressed as mean ± SEM. Statistics differences were analyzed by unpaired t test (**A-D**). P values are presented in the individual panels.

Figure 1A

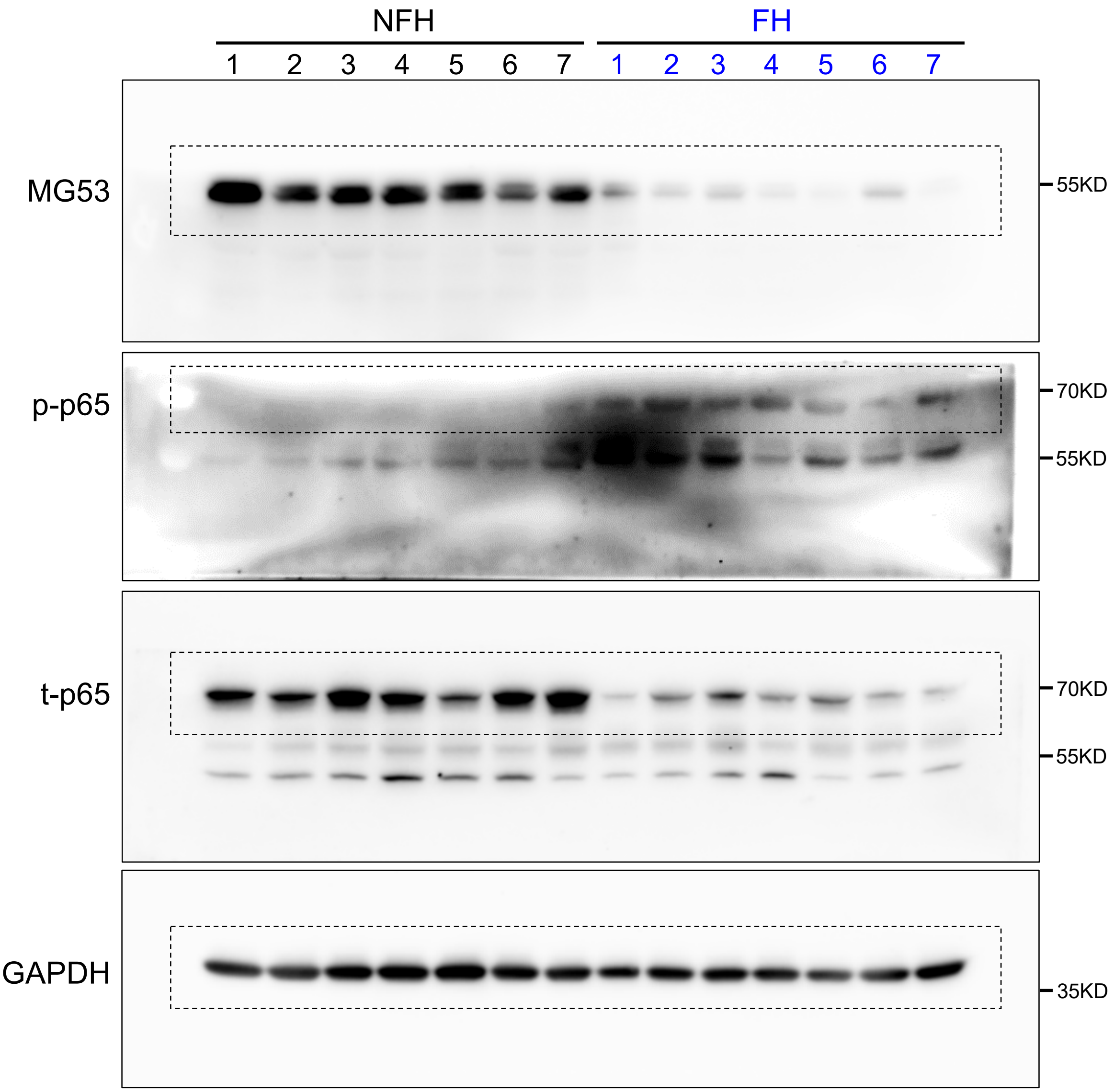
NFH
FH
1 2 3 4 5 6 7 1 2 3 4 5 6 7
MG53
p-p65
t-p65
GAPDH
55KD
70KD
55KD
70KD
55KD
35KD

Figure 1A (continued)

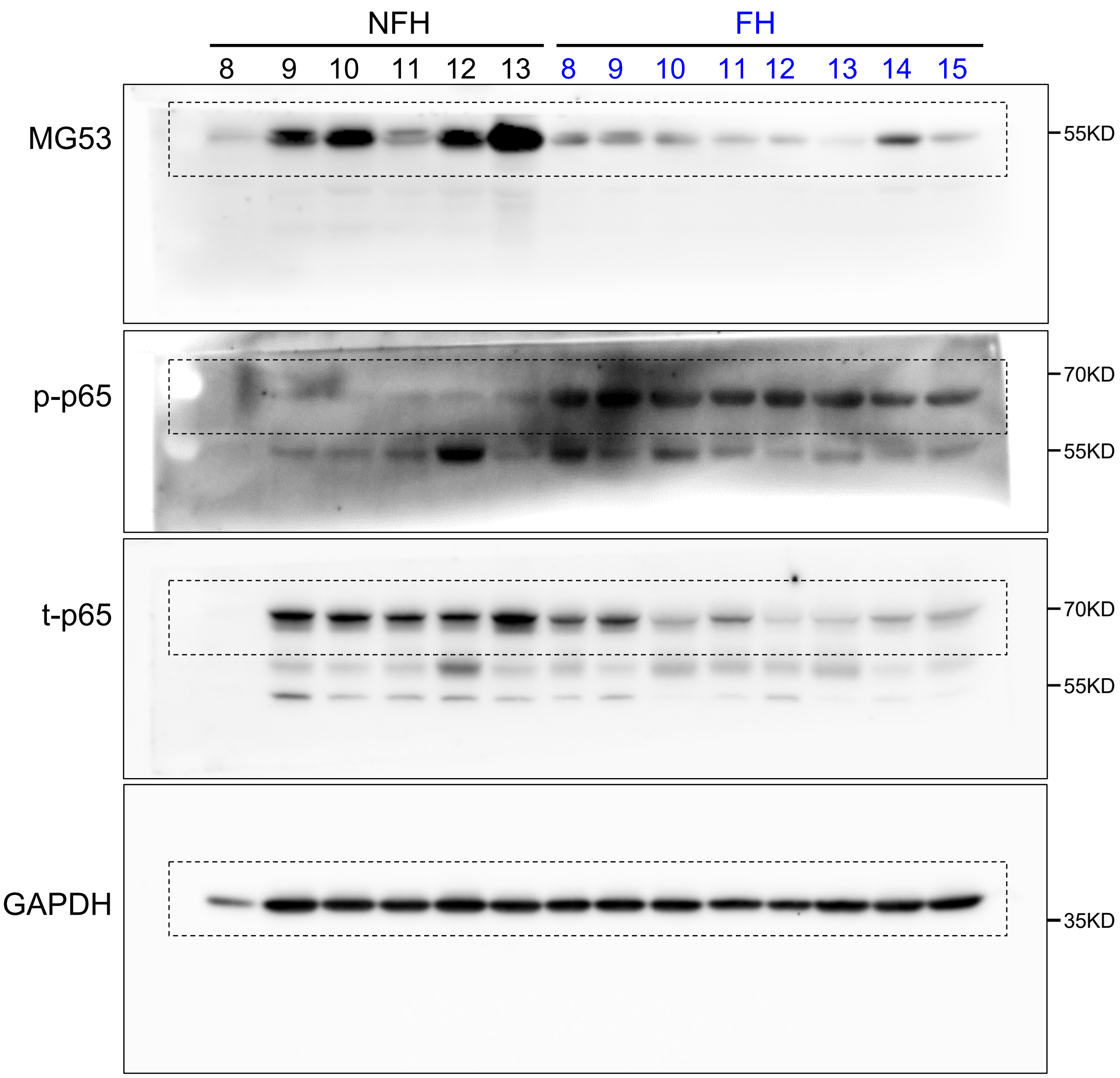

Figure 1E

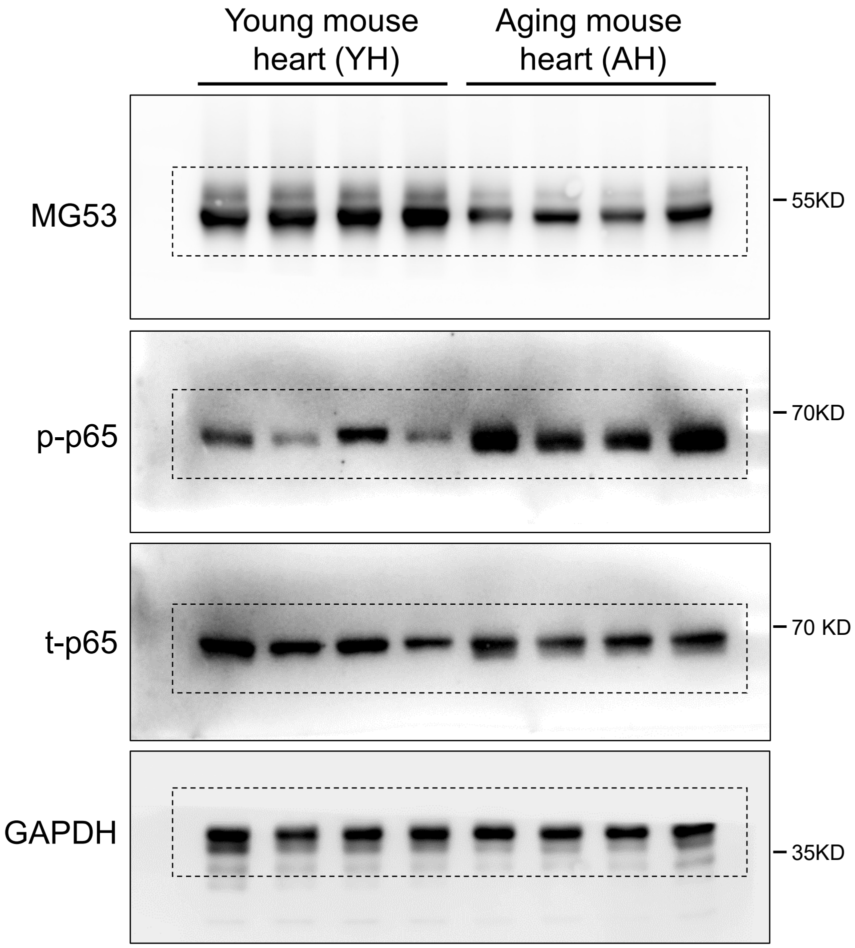
Young mouse heart (YH)
Aging mouse heart (AH)
MG53
p-p65
t-p65
GAPDH
55KD
70KD
70 KD
35KD

Figure 3B

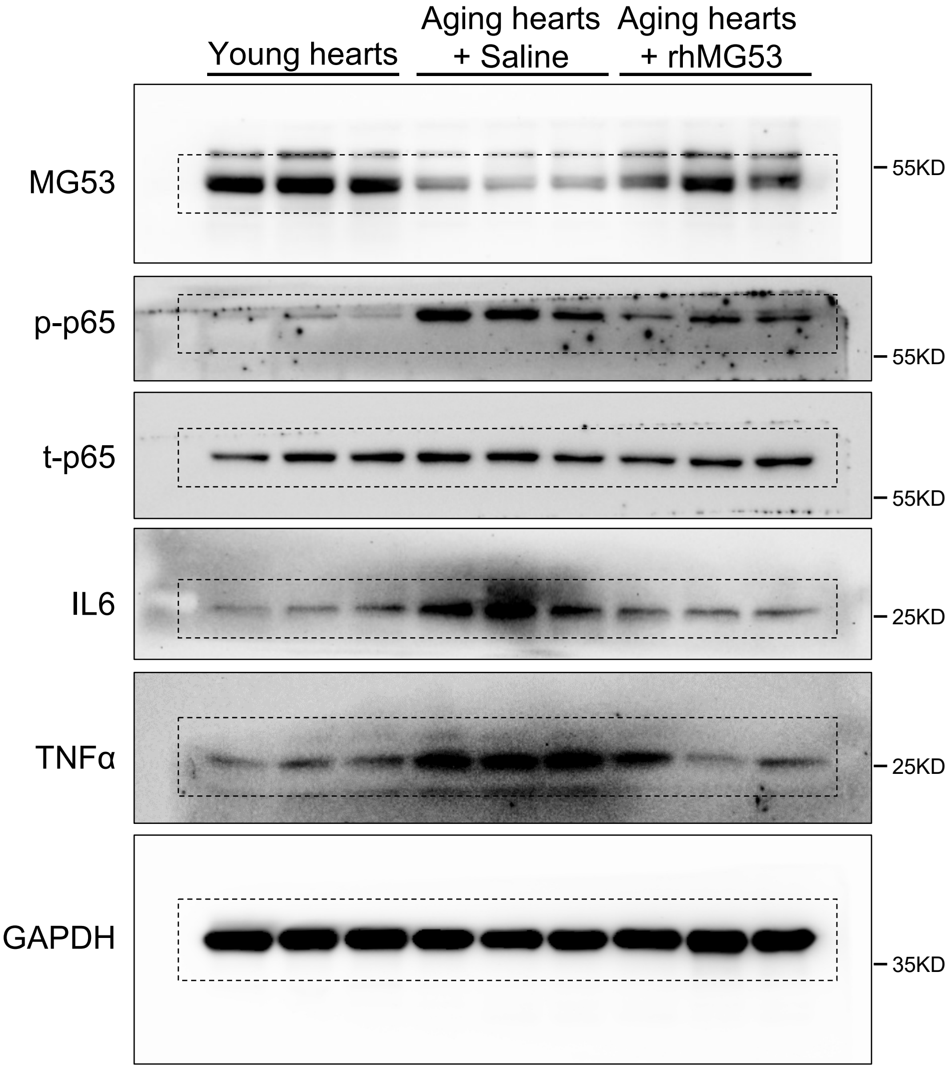

Young hearts
Aging hearts + Saline
Aging hearts + rhMG53
MG53
p-p65
t-p65
IL6
TNFα
GAPDH
55KD
55KD
55KD
25KD
25KD
35KD

Figure 4A

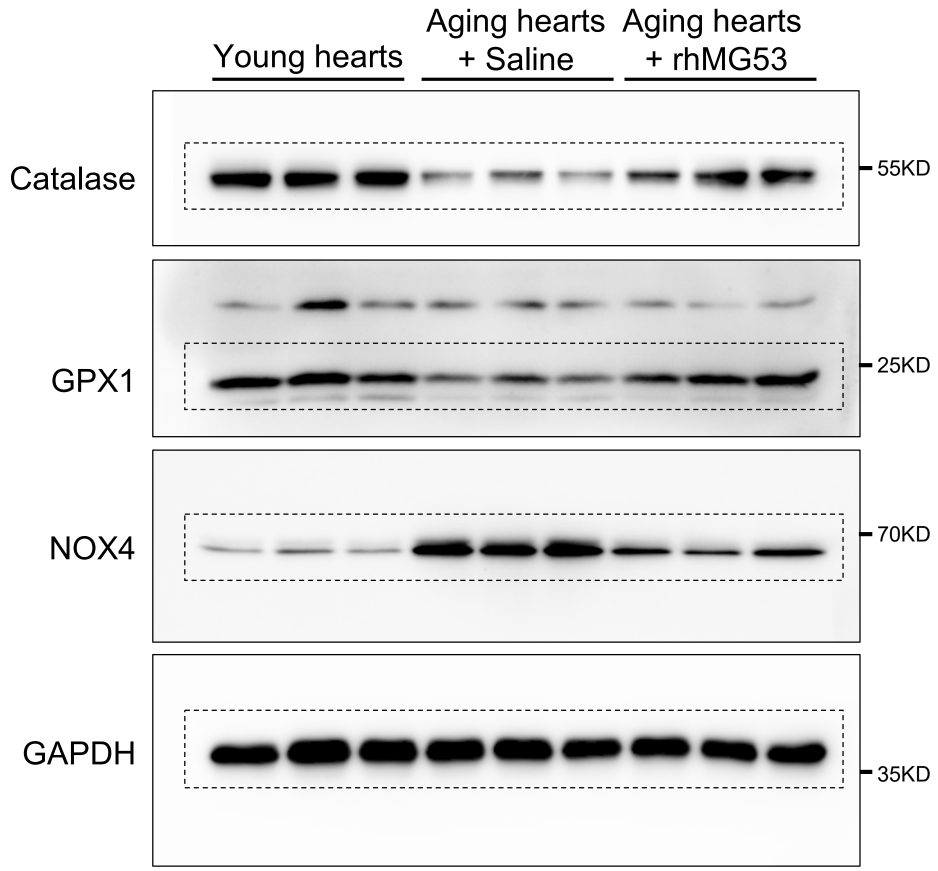

Young hearts
Aging hearts + Saline
Aging hearts + rhMG53
Catalase
GPX1
NOX4
GAPDH
55KD
25KD
70KD
35KD
